# Antibiotic stewardship and horizontal infection control are more effective than screening, isolation and eradication

**DOI:** 10.1007/s15010-018-1137-1

**Published:** 2018-05-23

**Authors:** S. W. Lemmen, K. Lewalter

**Affiliations:** Department of Infection Control and Infectious Diseases, Universtiy Hospital RWTH Aachen, 52074 Aachen, Germany

**Keywords:** Multidrug-resistant organisms, Screening, Isolation, Antibiotic stewardship, Hand hygiene, Antiseptic body wash

## Abstract

**Purpose:**

The global rise of multidrug resistant organisms (MDROs) is of major concern since infections by these pathogens are difficult, and in some cases, even impossible to treat. This review will discuss the effectiveness of a pathogen-independent alternative approach consisting of the implementation of antibiotic stewardship (ABS) programs, improvement of hand hygiene compliance, and daily antiseptic body washings instead of “screening, isolation and eradication” as recommended by many infection control guidelines today.

**Methods:**

A review of the literature.

**Results:**

The classical approach composed of screening, isolation and eradication has many limitations, including lack of standardization of the screening methods, risk of medical errors for patients in isolation and failure to eradicate resistant bacteria. Notably, concrete evidence that this current infection control approach actually prevents transmission is still lacking. We found that a novel approach with the training of infectious diseases specialists can reduce the usage of antimicrobials, thereby significantly decreasing the emergence of new MDROs. Moreover, increased hand hygiene compliance not only reduces transmission of MDROs, but also that of sensitive organisms causing the majority of nosocomial infections. Further, instruments, such as continuing education, bed-side observation, and the use of new tools, e.g. electronic wearables and Wi-Fi-equipped dispensers, are all options that can also improve the current low hand hygiene compliance levels. In addition, daily antiseptic body washes were observed to reduce the transmission of MDROs, especially those deriving from the body surface-like MRSA and VRE in specific settings. Finally, antiseptic body washes were seen to have similar effects on reducing transmission rates as screening and isolation measures.

**Conclusions:**

In summary, this review describes a novel evidence-based approach to counteract the growing medical challenge of increasing numbers of MDROs.

## Introduction

The global rise of multidrug-resistant organisms (MDROs), especially Gram-negative bacteria, poses a serious threat to patient safety due to limited therapeutic options. These options are further reduced by the recent discovery of a plasmid-mediated polymyxin resistance, generating almost pan-resistant bacteria [1]. Some of these pathogens derive from animal hosts and are transmitted to humans via the food chain, while others are imported from foreign countries, e.g., Asia or the Middle East [2]. However, the majority of MDROs will either be imported by local patients or be selected by antibiotics as well as partly transmitted in the hospital or health care facility itself.

This review will discuss an evidence-based alternative approach to the classical “screening, isolation, and eradication” one with a special emphasis on restricted usage of antibiotics, reduction of the bacterial burden of patients with antiseptics, and an increase in compliance with hand hygiene guidelines.

## Epidemiology

Methicillin-resistant *Staphylococcus aureus* (MRSA) is one of the leading MDRO and an important cause of health care-associated infections worldwide. MRSA was first discovered in the 1960s, and since then, it has become endemic in a vast array of health care settings in many countries around the world. Interestingly, however, after many years of steadily increasing, MRSA infection rates have started to decline in several countries. For example, in the European Union (EU), the mean percentage decreased from 18.8% in 2012 to 16.8% in 2015. This decrease could be partly due to the development of new antibiotics with an activity against MRSA in the last decades.

The prevalence of vancomycin-resistant enterococci (VRE) has remained almost unchanged in the EU with about 8% in 2015. However, in some countries, resistance rates are steadily increasing [3]. This trend is of concern since in comparison to MRSA, therapeutical options are currently even more limited. In contrast, resistance in Gram-negative bacteria caused by extended-spectrum ß-lactamases (ESBLs) is growing dramatically worldwide. Since these organisms are also often resistant to fluoroquinolones, antibiotic therapy options depend mainly on carbapenems. Thus, the worldwide increase in carbapenemase-producing strains is of major concern since there are no well-approved antimicrobial regimens currently available.

In a recent prospective multinational study, Grundmann et al. collected carbapenem non-susceptible isolates of *Klebsiella pneumoniae* and *Escherichia coli* from 455 hospitals in 36 EU-associated countries [4]. The prevalence of carbapenemase-producing organisms was on average 1.3 per 10,000 hospital admissions, with a range from 6 in Italy to 0.02 in Norway. 850/2301 (37%) of *K. pneumoniae* samples and 77/402 (19%) of *E. coli* samples were carbapenemase producers with KPC, NDM, OXA48, and VIM being the most common enzymes. The authors emphasize that although for most isolates alternative therapeutic options were available, resistance to all tested antibiotics was also reported in some cases.

In a systematic review and meta-analysis of 66 studies comprising 28,909 individuals, Karanika et al. determined the rate of fecal colonization with extended-spectrum ß-lactamase-producing enterobacteriaceae. The pooled worldwide prevalence of gut colonization was 14% (46% in Asia, only 4% in northern Europe, 6% in southern Europe) with a yearly increase of 5.4%. Main risk factors for colonization were antibiotic use within the past 4–12 months (RR = 1.6) and international travel (RR = 4) [5].

In Germany, recent data from the national nosocomial infections surveillance system (KISS) showed that approximately 3.5% of patients in intensive care units (ICUs) and 1.5% of patients on normal wards were tested MDRO-positive. The vast majority of these patients was colonized only and did not get an infection, e.g., 80% with MRSA and VRE and 50% with Gram-negative bacteria resistant to penicillins, cephalosporins, and quinolones [6]. In addition, according to the recent KISS data up to 80% of MDRO-positive patients were already colonized at the time of admission. Thus, nowadays, MDRO is no longer a predominantly hospital-associated problem, but more worrisomely, it is present in the ambulatory field and beyond.

## Preventative strategies

### Classical approach

#### Screening

In many infection control guidelines, swab-based screening for MDROs is recommended to identify asymptomatic carriers [7, 8]. Typical screening sites include nostrils, throat, and groin for MRSA and the perineal region for Gram-negative bacteria and VRE. However, important details concerning the best methodology are always lacking, e.g., which kind of swab to use, as there are many different materials available, e.g., rayon, polyurethane foam, or flocked nylon, which have a major impact on the detection rate. Warnke et al. compared different types of swabs for nasal MRSA screening in an artificial nose model. The best results were achieved for flocked nylon swabs with a sensitivity of 100%. Two kinds of rayon-made swabs only reached a sensitivity of 13 and 0%, respectively [9]. For Gram-negative bacteria, swabs made of nylon flocks or polyurethane foam were found to be superior to conventional rayon swabs (*p* < 0.001). In addition, swabbing deep intra-anally resulted in significantly higher recovery than perianal swabbing only (*p* < 0.001) [10]. Thus, the material and the structure of the swab itself have a major influence on the detection rate. However, neither are mentioned in infection control guidelines nor described in detail in most studies. In addition, the optimal frequency of the screening is also still under debate, e.g., entry screening, daily, weekly, or exit screening. Furthermore, different laboratory techniques can also be applied, e.g., normal culture, selective agars (e.g., chromogenic agar), or molecular techniques, and in the majority of the cases, the kind of technique is also not known to the clinician—although this can once again influence the detection rate.

Although all these limitations and pitfalls are obvious and known within the health care community, still infection control instruments, such as contact precautions with or without single room isolation, rely only on the screening results. Moreover, it is also still under debate which patient population should be screened: some medics demand universal screening, others try to define populations at risk. Finally, especially with a low detection rate of approximately only 1–2%, the cost–benefit ratio of screening is questioned in the literature [11].

In conclusion, due to the many different aspects, screening is very difficult to standardize and to the best of our knowledge so far not standardized. Therefore, false negative as well as false positive results are frequent. Thus, we advocate that screening should not be the major instrument used to assess a patient’s pathogen-specific colonization status.

#### Isolation

In most guidelines concerning the management of MDRO, contact precautions (CPs), including standard precautions plus isolation in a single room and usage of gowns and gloves are considered as essential infection control instruments to prevent transmission in a hospital setting. However, since most clinical studies investigating the effectiveness of these infection control measures have major deficiencies in design and reporting, evidence to support this approach is rather limited. On the other hand, one cannot exclude that well-designed prospective randomized controlled clinical studies might reveal a favorable effect.

In a prospective interrupted time series analysis in three general medical-surgical ICUs of two teaching hospitals in London, Cepeda et al. investigated the effect of moving or not moving MRSA-positive patients into single rooms to prevent MRSA transmission [12]. A cohort with 443 MRSA-positive patients was put into single room, and thus isolated; whereas, the other cohort with 423 patients was not. Both groups were screened regularly over a 6-month period. Interestingly, MRSA acquisition rates were similar for both cohorts. In detail, isolated and non-isolated patients had colonization rates of former negative patients of 12 and 10%, respectively. Compliance with hand hygiene was monitored during the study period and was estimated to be a very low 21% only. The authors conclude that in a setting of similar patient characteristics, MRSA acquisition rates could not be reduced by CPs alone, especially with a low compliance with hand hygiene.

In a systematic review and meta-analysis of nine studies, including a total of 30,949 patients, De Angelis et al. analyzed the effect of different infection control measures aimed at reducing the spread of VRE in a hospital setting. Notably, the rate of VRE acquisition was not reduced by CPs (RR = 1.08) at all; in contrast, the introduction of alcohol-based hand dispensers in place and an intensive educational program to improve compliance with hand hygiene reduced VRE acquisition significantly by 47%. [13]. In general, the overall quality of the single studies retracted was low and thus the results achieved by the meta-analysis is of limited evidence as well. Hence, a prospective randomized clinical trial is required to shed light on this particular matter.

Kullar et al. reviewed 15 studies questioning routine use of CPs especially for MRSA and conclude that there are only a few preliminary data to support this approach in endemic settings [14]. For example, Morgan et al. observed a total of 7743 health care worker (HCW) activities over 1989 h in four acute care facilities and documented that patients subjected to CPs had fewer HCW visits (− 36.4%, *p* < 0.001) as well as direct contacts (− 17.7%, *p* = 0.02) per hour [15]. In addition, significantly more alcoholic hand rubs (+ 15.8%, *p* = 0.001) were performed by HCWs on exiting an isolation room. The authors conclude that not CPs themselves but probably fewer visits and less direct contact of HCW with patients and especially better hand hygiene practices might be the reasons for the reduced risk of transmission.

Bardossy et al. investigated the effect of discontinuation CP in a retrospective study in an 800 bed teaching hospital. There were no significant differences in infection rates with MRSA and VRE catheter-associated urinary tract infections, ventilator-associated pneumonia, central-line associated bloodstream infections, surgical site infections and hospital-acquired MRSA bacteremia during the two 12-month periods including more than 76,000 patients [16]. However, asymptomatic transmission of MRSA and VRE were not investigated by routine screening. Martin et al. compared the laboratory-identified clinical culture rates of MRSA and VRE after cessation of CP and introduction of 2% chlorhexidine bathing in all units of two hospitals [17]. There was no significant difference in screening culture rates for MRSA (*p* = 0.09) and VRE (*p* = 0.14) before and after discontinuing of CP. Calculating the costs for nursing time spent with donning and gloving, costs for personal protective equipment and the chlorhexidine washing solution, annual savings of 643.776$ were achieved.

Furthermore, there is an increasing body of evidence showing that cessation of CPs does not lead to an increase in infection rates. In a quasi-experimental before-and-after study, Edmond et al. examined the effect of discontinuing CPs for MRSA and VRE in an 865-bed academic medical center [18]. During the study, CPs were replaced by hand hygiene promotion and daily antiseptic baths with chlorhexidine, and a bare-below-the-elbows protocol was implemented. Compliance with hand hygiene was high with over 85%; however, the compliance with the antiseptic bathing was not monitored. Comparing both strategies, no change was noticed in the rates of MRSA or VRE device-associated infections—in the ICUs as well as on the normal wards. In addition, no changes in catheter-associated urinary tract infections, central-line associated bloodstream infections, and ventilator-associated pneumonia were observed with all other pathogens.

Almyroudis et al. studied the effect of discontinuing systematic surveillance and CPs on the incidence of VRE bacteremia in a 125-bed hematology–oncology unit [19]. Between 2008 and 2011, the incidence of VRE bacteremia for patients under active surveillance and CPs was 2.32/1000 patient days (PD). Interestingly, from 2011 to 2014, surveillance and CPs were stopped and the incidence further decreased to 1.87/1000 PD. In 2013, daily bathing with chlorhexidine-impregnated washcloths was additionally implemented for all patients.

In addition, there are distinct disadvantages associated with the medical care of patients in isolation and these are of major concern and reported frequently. For example, Zahar et al. compared 170 patients in isolation with 980 non-isolated patients in two ICUs in France. Preventable adverse events, e.g., hypo- and hyperglycemia, errors in anticoagulant prescription, and ventilator-associated pneumonia due to resistant bacteria occurred significantly more frequent in isolation [20]. Patients reported more discomfort, depression, and anxiety when undergoing CPs and HCWs were less likely to visit patients as well as have less contact [21]. Compliance of HCWs with basic infection control guidelines is low, especially with high proportions of patients undergoing CPs [22].

In conclusion, with respect to the limited qualities and the diversity of the cited studies, active surveillance and CPs did not seem to prevent MRSA, VRE and ESBL transmission and infections in a susceptible patient population.

#### Eradication

In a prospective cohort study, Mattner et al. investigated the persistence of MRSA in 1032 MRSA-positive patients of a German university hospital between 2002 and 2005 [23]. Topical decolonization with mupirocin nasal ointment and antiseptic body wash with either octenidine or chlorhexidine for 5 days was performed, respectively. The overall half-time of MRSA persistence was 549 days and was even prolonged if multiple body sites were affected.

Ammerlaan et al. performed a systematic review concerning eradication of MRSA including 23 studies with a total of 2114 patients [24]. Nasal application of mupirocin for up to 7 days was most effective for MRSA eradication with an estimated success of 90% 1 week after treatment. However, in the long-term follow-up (14–365 days), recurrence of MRSA could be observed in about 40% of patients. The effectiveness of topical mupirocin treatment was further reduced when multiple body sites were colonized.

Eradication of VRE has been studied with different regimens of antimicrobial agents with high intraluminal concentrations, e.g., bacitracin, gentamicin or doxycycline; however, the results never showed a sustained decolonization due to these regimes [25, 26].

A novel approach with application of oral linezolid, daptomycin, and *Lactobacillus rhamnosus* following bowel preparation with polyethylene glycol achieved VRE clearance only in two of four liver transplant patients [27]. The duration of intestinal carriage of VRE is not well investigated, but is suspected to be even longer than MRSA.

The eradication of intestinal carriage of Gram-negative bacteria is even more difficult if not impossible. In a double-blind, randomized, placebo-controlled single center study, Huttner et al. investigated the efficacy of an oral decolonization regimen consisting of colistin, neomycin, and nitrofurantoin vs. placebo in a total of 54 patients with intestinal carriage of ESBL [28]. The primary outcome was detection of ESBL in rectal swabs after 28 days with additional cultures taken on day 6 of treatment and on days 1 and 7 after treatment. Regarding the primary outcome, there was no significant difference between both treatment groups [14/27 (52%) vs. 10/27 (37%), *p* = 0.27]. During treatment and on day 1 after treatment, intestinal carriage of ESBL was significantly lower in the treatment group. However, this effect was not observed on day 7 after treatment. Therefore, no long-term effect could be demonstrated. Thus, the usage of colistin for the purpose of decolonization as one of the last remaining effective antibiotics for highly resistant Gram-negative bacteria has to be questioned critically.

Finally, of note is that all other trials aiming to eradicate intestinal carriage of Gram-negative bacteria failed to show sustainable success [29].

Therefore, it is well accepted in the medical community that these pathogens cannot be successfully eradicated.

#### Summary of the classical approach

The complex pathogen-specific screening system as implemented in many medical institutions has many limitations since the swabbing material to be used, the frequency and the location patients should be screened are not well defined. In addition, neither the optimal quantity of the medical sample is known, nor is there any consistent laboratory standard to screen for the different MDROs. In summary, the current screening systems for complex pathogens that are in operation today are not standardized, nor can they be since multiple factors, especially sample size are not possible to standardize. In addition, the patient population that needs to be swabbed is not well defined, and thus, varies between medical institutions. So far, studies investigating the transmission rate of MDRO from patients undergoing CPs failed to find a reduction. However, it should also be considered that the study design was not always comprehensive in terms of patient numbers or pathogen monitoring, and therefore, the overall evidence is still preliminary at this stage. In contrast, disadvantages of single room isolation concerning patient discomfort, preventable adverse events, and reduced physician and HCWs contact time are well described. Finally, eradication, even for MRSA, is a major medical challenge, and currently, impossible in patients harboring VRE or multidrug-resistant Gram-negative bacteria. Thus, alternatives to current screening, isolating, and eradicating procedures are urgently required.

### Novel approach: known but neglected

#### Antibiotic stewardship programs (ABS)

In a recent systemic review and meta-analysis in which 32 out of 1113 identified articles from between 1960 and 2016 with more that 9 million patient days (pd) were included, Baur et al. could clearly demonstrate the influence of a restricted and rational usage of antimicrobials on the emergence of MDRO. With the implementation of the ABS program, infections and colonization with MRSA, multidrug-resistant Gram-negative bacteria, and *C. difficile* could be reduced significantly by 37, 51, and 32%, respectively. These interventions were especially effective in hematology–oncology departments with a 59% reduction of incidence rates: Probably to the low number of available papers, there was no effect on the incidence rates of VRE as well as quinolone- or aminoglycoside-resistant Gram-negative bacteria [30].

When ABS programs were combined with intensified infection control measures, the incidence rates of MDRO could be decreased by even up to 70% with hand hygiene compliance having the biggest effect of all the infection control measures [30]. Therefore, in many countries, infection control guidelines and strategies with a high level of evidence-based data have been proposed and implemented over the last decade to support rational use of antibiotics in hospitals and medical settings [31, 32].

In a recently published systemic review in the Cochrane Database, evidence with high-certainty was found that interventions due to an ABS program were effective in increasing compliance with local antibiotic policy and reducing duration of antibiotic treatment. Lower use of antibiotics probably did not increase mortality and likely reduced length of stay. However, there was too much variance in microbial outcomes to reliably assess the effect of change in antibiotic use concerning the emergency of resistance [33].

#### Hand hygiene

It is often described that a high compliance with hand hygiene, e.g., alcoholic hand rubs (AHR), is associated with a significant decrease in transmission of nosocomial pathogens, including MDROs. Already two decades ago, the pioneering work by Pittet et al. carried out at the university hospital of Geneva showed the significant effect on infection rates by increasing hand hygiene compliance [34]. In detail, they observed more than 20,000 opportunities for hand hygiene and due to intense education of their HCWs, compliance progressively and significantly improved from an initial 48% to 66% (*p* < 0.001) over a period from 1994 to 1997. At the same time, the overall prevalence of nosocomial infections dropped significantly from 16.9 to 9.9% (*p* = 0.04) and the transmission rates of MRSA were also reduced from 2.16 to 0.93 per 10,000 PD (*p* < 0.001) Importantly, this success as seen with better patient outcomes at this single Swiss medical center was also mirrored by others worldwide after also implementing better hand hygiene compliance guidelines via educational programs directed towards their HCWs [35].

However, compliance with hand hygiene guidelines is general low in many medical settings. In a prospective study, Scheithauer and colleagues compared direct observation vs. calculated disinfectant usage on a surgical (SICU), medical (MICU), and neurological ICU (NICU) [36]. During almost 300 h of observation, they documented a total of 1897 hand hygiene opportunities. Under observation, compliance rates were 39% for the SICU, 72% for the MICU, and 73% for the NICU. However, when compliance rates were calculated by disinfectant usage only, these dropped dramatically to 18% in the SICU, 26% in the MICU, and 22% in the NICU. In a prospective observational study, the same authors also documented almost 400 h of patient care in a SICU and MICU with 1727 hand hygiene indications for MRSA-positive patients and 1399 hand hygiene indications for ESBL-positive patients undergoing CPs with single room isolation [37]. Overall, compliance with hand hygiene was 49% with 47 and 43% for MRSA-positive patients and 54 and 51% for ESBL-positive patients in the SICU and MICU, respectively. Compliance rates were significantly higher after contact with patients and body fluids than before any contact with a patient, probably as a means of self-protection by the HCWs. Finally, gloves were used instead of hand hygiene indication 2 (before performing an aseptic task) in 38 and 47% of cases in the SICU and MICU, respectively.

A time saving approach is the implementation of a local champion as a role model, that is, a member of the team with a high compliance and taking over the responsibility to constantly remind others of proper hand hygiene [38]. In addition, direct observation with immediate feedback of an infection control nurse is the gold standard to monitor adherence and improve compliance with hand hygiene guidelines. However, this approach is labor intense, costly, and subject to the Hawthorne effect, i.e., participants may modify their behavior as a result of being part of an observational study. Therefore, novel techniques to overcome low hand hygiene compliance are urgently warranted. Such techniques include the usage of new electronic technologies, e.g., video-assisted observation, Wi-Fi-equipped dispensers, or wearable devices with digital automatic reporting options [39].

In conclusion, hand hygiene is regarded as the most effective single measure to prevent the transmission of nosocomial pathogens. However, maintaining hand hygiene compliance still requires ongoing education and training, motivation, and optimization of workflow. Key instruments to maintain high levels of hand hygiene compliance are direct observation and the use of new generations of electronic devices.

#### Antiseptics

Climo et al. conducted a multicenter, cluster-randomized crossover trial, which included 7727 patients from eight ICUs and a bone marrow transplant unit (BMTU). Daily bathing with 2% chlorhexidine (CHG) impregnated washcloths vs. usual care with soap reduced the transmission of both MRSA and VRE from 6.6 to 5.1/1000 pd significantly (*p* = 0.03). In addition, the rate of hospital-acquired bloodstream infections was reduced by 28% from 6.6 to 4.78/1000 pd (*p* = 0.007) [40]. Further, in a cluster-randomized trial, including more than 70,000 patients, Huang et al. documented a significant reduction in MRSA transmission and nosocomial bloodstream infections in patients after daily bathing with chlorhexidine combined with topical application of mupirocin [41].

Derde et al. performed an interrupted time series study and cluster-randomized trial with almost 9000 patients in different European ICUs. A high compliance with hand hygiene guidelines in combination with daily antiseptic body wash was observed to be effective in reducing colonization by resistant bacteria, particularly MRSA [42]. The Dutch authors conclude that “in the context of a sustained high level of compliance to hand hygiene and CHG bathing, screening and isolation of carriers do not reduce acquisition rates of multidrug-resistant bacteria”. In other words, screening and isolation had no additional effect on reducing colonization rates. Thus, the application of antiseptics in daily patient care in ICUs seems to be a promising alternative technique, especially when supported by a high level of compliance with hand hygiene guideline, even in the light of substantially lower rates of MDROs in Europe compared to the United States.

Finally, there is a legitimate concern about the emergence of chlorhexidine resistance. In this context, reduced susceptibility to chlorhexidine has already been observed for extremely drug-resistant strains of *Klebsiella pneumoniae*—a situation that thus needs to be further monitored [43]. A novel mechanism responsible for the adaption of *K. pneumonia* to CHG has recently been described as specific genetic mutations; in five out of six wild types these mutations led in addition to resistance to colistin [44]. On the other hand most of the CHG exposure of patients in the ICU would be explained by hand disinfectants or liquid soaps containing CHG [45].

In conclusion, there is valid and robust evidence that with a daily whole body wash with antiseptics in addition to standard infection control measurements, screening and isolation do not add any benefit. This body of evidence is derived from multiple well-designed randomized clinical trials, including more than 100,000 patients and from both the USA and Europe [40–42]. In addition, mathematical models show that universal decolonization is a cost-effective strategy to prevent MRSA transmission and infection in the endemic setting, especially for patients in ICUs [46]. However, reduction in susceptibility against CHG is of concern and should be monitored.

#### Transmission of MDROs in the hospital setting

In a recently published single center, longitudinal cohort study from England, transmission of *S. aureus* (Methicillin sensitive and Methicillin resistant) in an ICU was investigated [47]. Over a period of 14 months, 198 HCWs, 40 environmental locations and 1854 patients were sampled for the presence of *S. aureus*, a total of 1819 isolates was analyzed with whole genome sequencing (WGS). *S. aureus* was detected in 36.9% (73/198) of the HCWs, up to 32% of the environment and in 20% of the patients (371/1854). Routine infection control consisted of hand hygiene when indicated, daily antiseptic whole body washes, and once daily environmental disinfection. In general, contact precaution was not performed since the majority of the strains were oxacillin susceptible. In total, 25 identical pairs of strains were identified by WGS, resulting in a transmission rate of 1.3% (25/1819), only: 7 times from HCW, 2 times from the environment, and 16 times between patients. Although some important data, e.g., hand hygiene compliance and antibiotic consumption are lacking and the 4 weeks’ interval between the screenings might underestimate the role of HCW transient carriage, this study demonstrates the limited role of the environment and HCWs in transmitting *S. aureus* when a high standard of hygiene is present. Thus, a high compliance with standard precautions and hand hygiene should prevent transmission substantially.

In a retrospective study, Ford et al. investigated the risk of VRE transmission in a hematology–oncology unit [48]. From 2006 to 2014, 780 patients were reviewed, the majority of whom were accommodated in single rooms. After terminal disinfection, which was performed twofold, VRE was still detectable in 10% of the rooms. However, the rate of colonization was not increased in patients with a prior VRE-colonized room occupant (12.7 vs. 11.2 cases/1000 PD, *p* = 0.4). Molecular typing of 20 paired isolates from patients and prior occupants with PCR revealed identical strains in only 1 pair.

In an observational study at the University Hospital Basel, Switzerland, infection control experts investigated the transmission of ESBL-producing enterobacteriaceae without contact isolation [49]. From 1999 to 2011, a total of 93 ESBL-positive patients with 220 contact patients were included. The most common pathogens were *Escherichia coli* (73.1%) and *Klebsiella pneumoniae* (23.7%) and the mean exposure time to the index case was 4.3 days. A total of 133 screening samples from patients that shared the same room with a patient colonized or infected with ESBL-producing Gram-negative bacteria were analyzed. In only 1.5% of cases was a nosocomial pathogen transmission confirmed by molecular typing with pulsed field gel electrophoresis (PFGE). Thus, the authors stated that particularly with *E. coli*, nosocomial pathogen transmission without contact isolation is rare, and thus, a high level of standard infection precautions might be sufficient as an infection control standard in non-epidemic settings.

In contrast, Mitchell et al. stated in a systematic review and meta-analysis that the risk of acquiring an organism from prior room occupants is statistically increased [50]. They reviewed nine publications looking at different pathogens, i.e., MRSA, VRE, ESBL-producing Gram-negative bacteria, *Pseudomonas aeruginosa*, *Acinetobacter baumannii*, and *C. difficile*. They found that 6.2% (287/4643) of the patients acquired a pathogen from prior occupants, whereas only 3.2% (1112/34.886) of the patients admitted to unburdened rooms acquired one of the studied organisms. Eight out of nine studies applied phenotypical microbiological differentiation, only, and the one study in this review applying molecular typing (PFGE) found that occupying the same room concerning acquisition of ESBL-producing bacteria was not a significant risk factor [51].

In conclusion, transmission—independent of whether of Gram-positive or Gram-negative bacteria—is a rather rare event in hospitals in an endemic setting in contrast to outbreaks—and is often overestimated, especially with regard to MDROs. When molecular techniques are applied, different genotypes are mostly revealed. Thus, the benefit of implementing rather strict infection control strategies with the known disadvantages outlined above is questionable.

## Summary

Current epidemiological studies have shown a worldwide increase in resistant pathogens, especially in ESBL-producing Gram-negative bacteria having become the most common resistant pathogen worldwide and thus exceeded MRSA. The vast majority of patients with MDROs who are currently admitted to the hospital are already colonized or infected with these pathogens, so bacterial resistance is no longer a hospital-associated phenomenon. In addition, the vast majority of patients is and will remain colonized, only.

To date, the concept of “screening, isolation, and eradication” is recommended in many infection control guidelines. The aim is to detect MDRO early on to try to prevent its transmission, thereby reducing the bacterial burden. However, evidence to support this infection control strategy is still scarce and the results of many studies are inconclusive, and even, contradictory.

Concerning screening for pathogens, many questions are still a matter of medical debate, e.g., which swabs to be used, location and frequency, quantity of material required, and the laboratory technique to be applied. Moreover, screening is not at all standardized to date, despite being the major initial trigger for further infection control precautions. Further, the quality of the studies investigating the effects of CPs is very limited and the evidence that such measures prevent transmission is weak. However, medication errors, less time spend with patients, depression and anxiety of the patients due to isolation, as well as additional costs are well described. For MRSA, long persistence and a high recurrence rate after eradication are well documented and attempts to eradicate VRE or resistant Gram-negative bacteria are not even recommended.

Thus, an alternative regimen to this pathogen-specific “vertical approach” with a pathogen-independent “horizontal approach” is urgently needed to counter this ever-growing medical problem. Antibiotics, known to be the major driver and trigger for MDROs, are still overused and partly abused, e.g., prolonged surgical site prophylaxis, treatment of asymptomatic bacteriuria, or upper respiratory infections. A limited and restricted usage of antimicrobials has been requested from different societies all over the world for decades. There is overwhelming evidence that by implementing ABS programs, the prevalence of MDROs will be reduced significantly. Thus, additional infectious disease and infection control specialists are urgently needed to address this pressing medical issue and to help better implement the required measures aimed at solving this worldwide and ever-growing pathogen problem.

It is well accepted that hand hygiene compliance is the major single infection control measure that is effective in preventing transmission. However, compliance is still surprisingly low and action needs to be taken so that it is increased and stays increased. Note that clean hands do not only reduce transmission of MDROs, but also of sensitive organisms that can cause more than 95% of nosocomial infections. Thus, continuing education, bedside observation, and new tools, such as electronic wearables and Wi-Fi-equipped dispensers, are all options to help improve this current lack of compliance.

Finally, full and daily body washes with antiseptics did show in specific settings, e.g., ICUs and a BMTU, a reduction in the transmission of MDROs, especially deriving from the body surface, e.g., MRSA and VRE. In well-designed studies, antiseptic body washes were observed to have similar effects compared to screening and isolation measures. However, the possible emergence of reduced susceptibility to antiseptics such as chlorhexidine must also be considered.

In conclusion, instead of the popular current “screening, isolation, and eradication” approaches, we advocate to increase the effort to train infectious disease and infection control specialists with the implementation of ABS programs, to set up better hand hygiene compliance regimes with monitoring to maintain a high level of compliance as well as to perform daily antiseptic body washes in a specific patient populations (Table 1).

**Table 1:** Advantages and disadvantages of the classical versuch the novel approach

	Advantages	Ref.	Disadvantages	Ref.
Classical approach				
Screening	Adherence to infection control guidelines Detection of asymptomatic carriers Following political, public and patient requests and concerns In case of accusation: proof that patients were already colonized when admitted	[7, 8] [7, 8]	No international/national standard Limited sensitivity Costly benefit ratio questionable Arbitrary definition between ambulant (< 72 h) and nosocomially (> 72 h) acquired	[9, 10] [9, 10] [11] [7, 8]
Eradication	Decrease of risk of transmission by eradication Decreased risk of infection	[23, 24]	High recurrence rate in up to 40% of MRSA Failure to achieve eradication in VRE and Gram-negative bacteria Usage of colistin in many eradication regimen as “last resort antibiotic”	[23, 24] [25–29] [28, 29]
Isolation	Adherence to infection control guidelines Well accepted as infection control tool in outbreak setting Well investigated in a bundle approach to prevent transmission	[7, 8] [7, 8] [7, 8]	No reduction in colonization and infection rates Less visits and less direct contact time Discontinue had no adverse impact on infection rates Increase in preventable adverse events and medical errors Isolated patients experience more anxiety, depression and fear Compliance with standard infection control decreases when many patients are in CP Stigmatizing of patients in isolation Extra costs for personal protective equipment	[12, 13] [15] [15, 21] [18] [19] [20] [20] [21] [22]
Novel approach				
Antibiotic stewardship	Significant reduction in MDRO Significant reduction of CDI Adherence to guidelines with high level of evidence Increase of compliance with local antibiotic policy Reduction in duration of antibiotic therapy Cost saving due to less antibiotic usage	[30] [30] [31, 32]	Limited numbers of infectious disease specialists available in some countries (e.g., Germany) Additional personnel costs	[31, 32] [31, 32]
Antiseptic washing	Significant reduction of transmission of MRSA and VRE in ICU and BMT Significant reduction of BSI in ICU As an horizontal approach effective for sensitive pathogen, as well Time saving in comparison to washing with water and soap	[40] [40, 41] [41]	Concern of emergence of resistance against CHG Reduced susceptibility against CHG has been observed Different mechanisms of reduced susceptibility in bacteria has been described Adverse events	[43, 45] [43] [44] [40, 41]
Hand hygiene	Reduction of transmission of MDRO Reduction of infection rates As an horizontal approach effective for sensitive pathogen, as well Additional personnel protection against potential infectious pathogen	[34, 35]	In general, low compliance Additional time, commitment and effort requested to improve compliance New equipment (e.g. Wi-Fi surveillance, wearables) require technical and financial support	[36, 37] [34, 35, 38] [39]

## References

[1] Liu YY, Wang Y, Walsh TR (2016). Emergence of plasmid-mediated colistin resistance mechanism MCR-1 in animals and human beings in China: a microbiological and molecular biology study. Lancet Inf Dis.

[2] Hawkey PM (2015). Multidrug-resistant Gram-negative bacteria: a product of globalization. J Hosp Infect.

[3] https://ecdc.europa.eu/sites/portal/files/media/en/publications/Publications/antimicrobial-resistance-europe-2015.pdf. (Accessed 12 Sep 2017).

[4] Grundmann H, Glasner C, Albiger B (2017). Occurence of carbapenemase-producing *Klebsiella pneumoniae* and *Escherichia coli* in the European survey of carbapenemase-producing enterobacteriaceae (EuSCAPE): a prospective, multinational study. Lancet Inf Dis.

[5] Karanika S, Karantanos T, Arvanitis M (2016). Fecal colonization with extended-spectrum beta-lactamase-producing enterobacteriaceae and risk factors among healthy individuals: a systematic review and metaanalysis. Clin Infect Dis.

[6] Geffers C, Maechler F, Behnke M (2016). Multiresistente Erreger, Epidemiologie, Surveillance und Bedeutung. Anästhesiol Intensivmed Notfallmed Schmerzther.

[7] Recommendations for prevention and control of methicillin-resistant staphylococcus aureus (MRSA) in medical and nursing facilities. Bundesgesundheitsbl 2014;57:696–732. 24987771

[8] Tacconelli E, Cataldo MA, Dancer SJ (2014). ESCMID guidelines for the management of the infection control measures to reduce transmission of multidrug-resistant Gram-negative bacteria in hospitalized patients. Clin Microbiol Infect.

[9] Warnke P, Frickmann H, Ottl P (2014). Nasal screening for MRSA: different swabs—different results!. PLoS One.

[10] Warnke P, Pohl FPJ, Kundt G (2016). Screening for Gram-negative bacteria: impact of preanalytical parameters. Sci Rep.

[11] Robotham JV, Deeny SR, Fuller C (2016). Cost-effectiveness of national mandatory screening of all admissions to English National Health Service hospitals for methicillin-resistant Staphylococcus aureus: a mathematical modelling study. Lancet Inf Dis.

[12] Cepeda JA, Whitehouse T, Cooper B (2005). Isolation of patients in single rooms or cohorts to reduce the spread of MRSA in intensive-care units: prospective two-centre study. Lancet.

[13] De Angelis G, Cataldo MA, De Waure C (2014). Infection control and prevention measures to reduce the spread of vancomycin-resistant enterococci in hospitalized patients: a systematic review and meta-analysis. J Antimicrob Chemother.

[14] Kullar R, Vassallo A, Turkel S (2016). Degowning the controversies of contact pre-cautions for methicillin-resistant *Staphylococcus aureus*: a review. Am J Infect Control.

[15] Morgan DJ, Pineles L, Shardell M (2013). The effect of contact precautions on healthcare worker activity in acute care hospitals. Infect Control Hosp Epidemiol.

[16] Bardossy AC, Alsafadi MY, Starr P (2017). Evaluation of contact precautions for methicillin-resistant *Staphylococcus aureus* and vancomycin-resistant Enterococcus. Am J Infect Control.

[17] Martin EM, Russell D, Rubin Z (2016). Elimination of routine contact precautions for endemic methicillin-resistant *Staphylococcus aureus* and vancomycin-resistant Enterococcus: a retrospective quasi-experimental study. Infect Control Hosp Epidemiol.

[18] Edmond MB, Masroor N, Stevens MP (2015). The impact of discontinuing contact precautions for VRE and MRSA on device-associated infections. Infect Control Hosp Epidemiol.

[19] Almyroudis NG, Osawa R, Samonis G (2016). Discontinuitation of systematic surveillance and contact precautions for vancomycin-resistant enterococcus (VRE) and its impact on the incidence of VRE faecium bacteremia in patients with hematologic malignancies. Infect Control Hosp Epidemiol.

[20] Zahar JR, Garrouste-Orgeas M, Vesin A (2013). Impact of contact isolation for multi-drug-resistant organisms on the occurrence of medical errors and adverse events. Intensive Care Med.

[21] Morgan DJ, Diekema DJ, Sepkowitz K (2009). Adverse outcomes associated with contact precautions: a review of the literature. Am J Infect Control.

[22] Dhar S, Marchaim D, Tansek R (2014). Contact precautions: more is not necessarily better. Infect Control Hosp Epidemiol.

[23] Mattner F, Biertz F, Ziesing S (2010). Long-term persistence of MRSA in re-admitted patients. Infection.

[24] Ammerlaan HSM, Kluytmans JAJW, Wertheim HFL (2009). Eradication of methicillin-resistant *Staphylococcus aureus* carriage: a systematic review. Clin Inf Dis.

[25] Weinstein MR, Dedier H, Brunton J (1999). Lack of efficacy of oral bacitracin plus doxycycline for the eradication of stool colonization with vancomycin-resistant *Enterococcus faecium*. Clin Infect Dis.

[26] Hachem R, Raad I (2002). Failure of oral antimicrobial agents in eradicating gastrointestinal colonization with vancomycin-resistant enterococci. Infect Control Hosp Epidemiol.

[27] Cheng VC, Chen JHK, Tai JWM (2014). Decolonization of gastrointestinal carriage of vancomycin-resistant *Enterococcus faecium*: case series and review of the literature. BMC Infect Dis.

[28] Huttner B, Haustein T, Uckay I (2013). Decolonization of intestinal carriage of extended-spectrum β-lactamase-producing enterobacteriaceae with oral colistin and neomycin: a randomized, double-blind, placebo-controlled trial. J Antimicrob Chemother.

[29] Saidel-Odes L, Polachek H, Peled N (2012). A randomized, double-blind, placebo-controlled trial of selective digestive decontamination using oral gentamicin and oral polymyxin E for eradication of carbapenem-resistant *Klebsiella pneumoniae* carriage. Infect Control Hosp Epidemiol.

[30] Baur D, Gladstone BP, Burkert F (2017). Effect of antibiotic stewardship on the incidence of infection and colonization with antibiotic-resistant bacteria and Clostridium difficile infection: a systematic review and meta-analysis. Lancet Inf Dis.

[31] De With K, Allerberger F, Amann S (2016). Strategies to enhance rational use of antibiotics in hospital: a guideline by the German Society for Infectious Diseases. Infection.

[32] Barlam TF, Cosgrove SE, Abbo LM (2016). Implementing an antibiotic stewardship program: guidelines by the Infectious Diseases Society of America and the Society for Healthcare Epidemiology of America. Clin Infect Dis.

[33] Davey P, Marwick CA, Scott CL (2017). Interventions to improve antibiotic prescribing practices for hospital inpatients. Cochrane Database Syst Rev.

[34] Pittet D, Hugonnet S, Harbarth S (2000). Effectiveness of a hospital-wide program to improve compliance with hand hygiene. Lancet.

[35] Sroka S, Gastmeier P, Meyer E (2010). Impact of alcohol hand-rub use on methicillin-resistant *Staphylococcus aureus*: an analysis of the literature. J Hosp Infect.

[36] Scheithauer S, Haefner H, Schwanz T (2009). Compliance with hand hygiene on surgical, medical, and neurologic intensive care units: direct observation versus calculated disinfectant usage. Am J Infect Control.

[37] Scheithauer S, Oberröhrmann A, Haefner H (2010). Compliance with hand hygiene in patients with methicillin-resistant *Staphylococcus aureus* and extended-spectrum β-lactamase-producing enterobacteria. J Hosp Infect.

[38] Pan SC, Tien KL, Hung IC (2013). Compliance of health care workers with hand hygiene practices: independent advantages of overt and covert observers. PLoS One.

[39] Ellingson K, Haas JP, Aiello AE (2014). Strategies to prevent healthcare-associated infections through hand hygiene. Infect Control Hosp Epidemiol.

[40] Climo MW, Yokoe DS, Warren DK (2013). Effect of daily chlorhexidine bathing on hospital-acquired infection. N Engl J Med.

[41] Huang SS, Septimus E, Kleinman K (2013). Targeted versus universal decolonization to prevent ICU infection. N Engl J Med.

[42] Derde LPG, Cooper BS, Goosens H (2014). Interventions to reduce colonization and transmission of antimicrobial-resistant bacteria in intensive care units: an interrupted time series study and cluster randomized trial. Lancet Inf Dis.

[43] Naparstek L, Carmenli Y, Chmelnitsky I (2012). Reduced susceptibility to chlorhexidine among extremely-drug-resistant strains of *Klebsiella pneumoniae*. J Hosp Inf.

[44] Wand ME, Bock LJ, Bonney LC, et al. Mechanisms of increased resistance to chlorhexidine and cross-resistance to colistine following exposure of *Klebsiella pneumoniae* clinical isolates to chlorhexidine. Antimicrob Agents Chemother. 2016. 10.1128/aac.01162-16. 10.1128/AAC.01162-16 PMC5192135 27799211

[45] Kampf G (2016). Acquired resistance to chlorhexidine—is it time to establish an “antiseptic stewardship” initiative?. J Hosp Infect.

[46] Gidengil CA, Gay C, Huang SS (2015). Cost-effectiveness of strategies to prevent methicillin-resistant *Staphylococcus aureus* transmission and infection in an intensive care unit. Infect Control Hosp Epidemiol.

[47] Price JR, Cole K, Bexley A (2017). Transmission of *Staphylococcus aureus* between health-care workers, the environment, and patients in an intensive care unit: a longitudinal cohort study based on whole-genome sequencing. Lancet Inf Dis.

[48] Ford CD, Lopansri BK, Gazdik MA (2016). Room contamination, patient colonization pressure and the risk of vancomycin-resistant Enterococcus colonization on a unit dedicated to the treatment of hematologic malignancies and hematopoetic stem cell transplantation. Am J Infect Control.

[49] Tschudin-Sutter S, Frei R, Dangel M (2012). Rate of transmission of extended-spectrum beta-lactamase-producing enterobacteriaceae without contact isolation. Clin Inf Dis.

[50] Mitchell BG, Dancer SJ, Anderson M (2015). Risk of organism acquisition from prior room occupants: a systematic review and meta-analysis. J Hosp Infect.

[51] Ajao AO, Johnson JK, Harris AD (2013). Risk of acquiring extended-spectrum β-lactamase-producing *Klebsiella* species and *Escherichia coli* from prior room occupants in the intensive care unit. Infect Control Hosp Epidemiol.

